# *Mycobacterium abscessus* Pulmonary Disease in a Myasthenia Gravis Patient: A Case Report

**DOI:** 10.1002/rcr2.70166

**Published:** 2025-07-14

**Authors:** Daisy Lu, Steven Y. C. Tong, Belinda Cruse, Kasha P. Singh, Justin T. Denholm, Megan Rees

**Affiliations:** ^1^ Department of Respiratory and Sleep Disorders Medicine Royal Melbourne Hospital Melbourne Victoria Australia; ^2^ Victorian Infectious Diseases Service Royal Melbourne Hospital, at the Peter Doherty Institute for Infection and Immunity Melbourne Victoria Australia; ^3^ Department of Infectious Diseases Peter Doherty Institute for Infection and Immunity, University of Melbourne Melbourne Victoria Australia; ^4^ Department of Neurology Royal Melbourne Hospital Melbourne Victoria Australia; ^5^ Department of Medicine Royal Melbourne Hospital, University of Melbourne Melbourne Victoria Australia

**Keywords:** bronchiectasis, Myasthenia Gravis, *Mycobacterium abscessus*, non‐tuberculous mycobacteria

## Abstract

There is limited guidance on managing *Mycobacterium abscessus* pulmonary disease in Myasthenia Gravis patients. Macrolides and aminoglycosides form the backbone of *M. abscessus* pulmonary disease treatment but are avoided in Myasthenia Gravis patients due to the risk of precipitating myasthenic crisis. A 29‐year‐old female, with a history of anti‐acetylcholine receptor antibody‐positive Myasthenia Gravis and bronchiectasis, was diagnosed with *M. abscessus* pulmonary disease. She was commenced on a macrolide‐based regime safely with close monitoring, pretreatment with intravenous immunoglobulin (1 g/kg),10 mg prednisolone, 500 mg rituximab and augmentation of the neuromuscular junction with pyridostigmine and 3,4‐Diaminopyridine. She was safely discharged to the hospital in the home program and achieved clinical, radiological and microbiological response.

## Graphical abstract

Reports on *Mycobacterium abscessus* complex (MABC) pulmonary disease and Myasthenia Gravis (MG) are rare; to our knowledge, this is the second case describing successful treatment of MABC pulmonary infection in this population and the first with current guideline‐directed therapy. Our case highlights that guideline‐directed treatment of MABC pulmonary disease with macrolide‐based regimes can be safely initiated in patients with MG, provided close clinical monitoring, optimisation of neuromuscular function and multidisciplinary care.

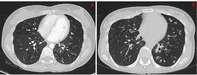

## 1 Introduction

There are unique issues in treating *Mycobacterium abscessus* complex (MABC) infections in patients with Myasthenia Gravis (MG). Macrolides and aminoglycosides, which form the backbone of treatment, affect the neuromuscular junction and recommendations are to avoid them in MG [1]. We share a case of successful initiation of macrolide and aminoglycoside‐based treatment for MABC pulmonary disease in a patient with MG.

## 2 Case Report

A 29‐year‐old female, with a history of anti‐acetylcholine receptor antibody‐positive MG and bronchiectasis, presented for assessment of new respiratory decline. Prior MG management included a thymectomy in 2011, mycophenolate and azathioprine from 2011 to 2018, intravenous immunoglobulin (IVIG) and rituximab since 2018, and a previous myasthenic crisis requiring intensive care admission in 2018.

**FIGURE 1:**
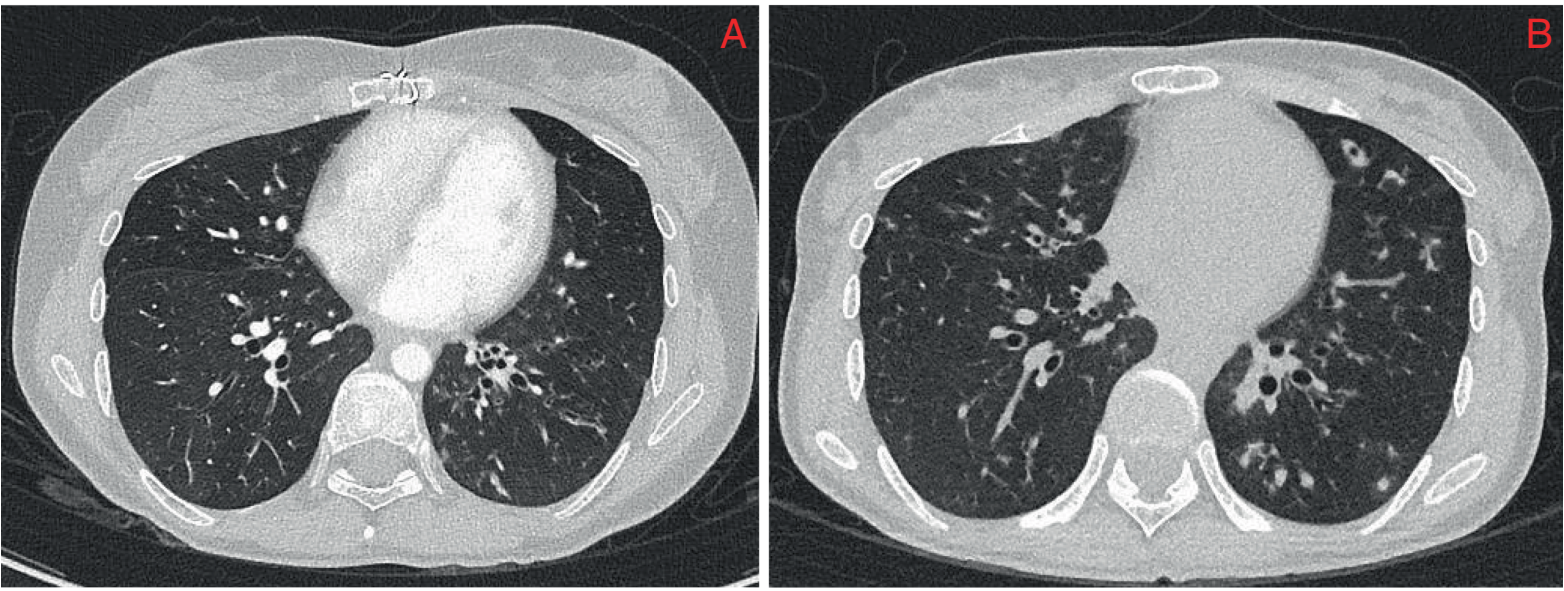
Computed tomography (CT). (A) Chest CT in 2020 showing mild bronchiectasis at the left base. (B) Chest CT in 2022 showing progression of the patient's bronchiectasis with increased nodularity and bronchial wall thickening.

In 2020, she was referred to the infectious diseases clinic with 3 months of occasional productive cough and repeated sputum sampling that isolated MABC. A chest computed tomography (CT) showed mild bronchiectasis in the left base (Figure 1A). After 2 years of clinical and radiological stability, she was referred to the respiratory clinic with 2 months of persistent productive cough and weight loss. A repeat CT revealed significant progression of her bronchiectasis with increased nodularity and bronchial wall thickening (Figure 1B). MABC was isolated in four sputum samples, and she was diagnosed with MABC pulmonary disease. Our centre could not determine subspecies; susceptibilities were performed by drug susceptibility testing, including assessment for inducible macrolide resistance, which demonstrated sensitivity to amikacin, clarithromycin and linezolid, intermediate sensitivity to doxycycline, cefoxitin and moxifloxacin and resistance to ciprofloxacin, cotrimoxazole and imipenem, with a tigecycline minimum inhibitory concentration of 0.5 μg/mL. Lung function testing demonstrated severe restriction on spirometry with gas transfer in the normal predicted range when accounting for alveolar volume; results consistent with neuromuscular dysfunction. Baseline audiology assessment, regular chest physiotherapy and daily bedside forced vital capacity (FVC) measurements were completed; At baseline, FVC was 1.29 L, but there were no other clinical manifestations of her MG, with an MG composite score of 0.

She was pretreated with IVIG induction (1 g/kg),10 mg prednisolone, 500 mg rituximab and was electively admitted to a tertiary centre for commencement of MABC treatment. She commenced on amikacin 15 mg/kg IV once daily and tigecycline 50 mg IV twice daily via a peripherally inserted central catheter, clofazimine 50 mg orally daily and clarithromycin 500 mg orally twice daily. With initiation of antibiotics, 60 mg pyridostigmine three times daily was commenced.

On commencement of therapy, she developed significant MG symptoms, including fatigable limb and neck weakness. This settled with the up‐titration of pyridostigmine to 60 mg four times daily, addition of 3,4‐Diaminopyridine (10 mg four times daily, subsequently reduced to 5 mg every 4 h due to intolerance). We also replaced clarithromycin with 500 mg azithromycin daily on Day 10 due to nausea. Amikacin was ceased on day eight due to vestibular toxicity. She was discharged home to the Outpatient Parenteral Antimicrobial Therapy service on Day 23 and completed 4 weeks of parenteral intensive phase treatment, followed by an oral maintenance phase with daily doses of clofazimine 50 mg, azithromycin 500 mg and linezolid 600 mg. Her productive cough resolved 2 months after commencement of therapy. MG medications were slowly weaned over the next 3 months. Treatment was intended for 12 months but discontinued after 7 months due to good clinical response and the interval development of peripheral neuropathy, which resolved off treatment. After initiation of therapy, she ceased producing sputum, and a follow‐up sputum culture was not performed while on treatment. After completion of therapy, she remained well, and an induced sputum collected 13 months after treatment initiation was smear and culture negative for MABC.

## 3 Discussion

MABC pulmonary disease is notoriously challenging to treat due to intrinsic and acquired multidrug resistance. Macrolide therapy is critical for successful MABC management, with high rates of treatment failure in macrolide resistance [2]. Guidelines suggest a macrolide‐containing regimen with at least three antimicrobials with in vitro susceptibility, with an intensive parenteral phase followed by a long continuation phase [2]. However, current advice is to avoid all macrolides as these can worsen MG symptoms, including precipitating myasthenia crisis [1].

To reduce the risk and severity of an acute MG exacerbation, our patient was pre‐treated with immunomodulatory therapies (IVIG, rituximab and moderate‐dose prednisolone), and postsynaptic function was optimised with pyridostigmine; all treatments recommended in symptomatic MG in addition to nonpharmacological therapy and close monitoring [3]. Noting the evidence suggesting macrolides may impair neurotransmission at the presynaptic level, 3,4‐Diaminopyridine was added upon the first signs of neuromuscular deterioration [1], allowing recommencement of azithromycin without deterioration. Further complicating treatment was the development of vestibular toxicity with amikacin, necessitating its cessation on Day 8.

Considering the existing literature, a Japanese case by Matsuse et al. described outpatient management using oral clarithromycin, levofloxacin and faropenem in a patient with MG and MABC pulmonary infection with mild clinical symptoms and chest radiological findings [4]. Our case detailed the treatment of a more severe case of MABC pulmonary disease with an initial intensive parenteral and subsequent oral maintenance phase. Imoto et al. described a case of disseminated *M. abscessus* subs. *massiliense* infection in a patient with Good syndrome and MG [5]; They used a combination of azithromycin, levofloxacin, imipenem/cilastatin, minocycline and linezolid. Despite this, the infection progressed and the patient died on Day 49.

Reports on MABC pulmonary disease and MG are rare; to our knowledge, this is the second case describing successful treatment of MABC pulmonary infection in this population and the first with current guideline‐directed therapy. Our case highlights that guideline‐directed treatment of MABC pulmonary disease with macrolide‐based regimes can be safely initiated in patients with MG, provided close clinical monitoring, optimisation of neuromuscular function and multidisciplinary care.

## Author Contributions

All authors qualified for authorship. Authors' main contributions regarding this manuscript are as follows. Daisy Lu: drafting the draft. Steven Y.C. Tong, Belinda Cruse, Kasha P. Singh, Justin T. Denholm, Megan Rees: supervision, revising the draft, and final approval of the draft.

## Ethics Statement

The authors declare that written informed consent was obtained for the publication of this manuscript and accompanying images using the form provided by the Journal.

## Conflicts of Interest

The authors declare no conflicts of interest.

## Data Availability

Data sharing not applicable to this article as no datasets were generated or analysed during the current study.
